# EFFECTIVENESS OF A TECHNOLOGICAL PLATFORM TO MANAGE FRAILTY: RESULTS FROM THE POSITIVE AND INTEGRACAM STUDIES

**DOI:** 10.1093/geroni/igae098.1278

**Published:** 2024-12-31

**Authors:** Leocadio Rodriguez-Manas, Alba Maria Costa Grille, Irene Molina Hermosilla, Irene Alfonso Lopez, Rodrigo Perez-Rodriguez, Ignacio Peinado-Martinez, Elena Villalba-Mora

**Affiliations:** Hospital Universitario de Getafe, Getafe, Madrid, Spain; Hospital Universitario de Getafe, Getafe, Madrid, Spain; Hospital Universitario de Getafe, Getafe, Madrid, Spain; Hospital Universitario de Getafe, Getafe, Madrid, Spain; Universidad Rey Juan Carlos, Fuenlabrada, Madrid, Spain; Hospital Universitario de Getafe, Getafe, Madrid, Spain; Universidad Politecnica de Madrid, Pozuelo de Alarcon, Madrid, Spain

## Abstract

Technologies can assist in managing frailty through home monitoring and intervention systems, though evidence of their clinical benefit is lacking. Our study had two components. A) A two-arm (Intervention vs. Control) RCT named POSITIVE (NCT04592146) with a 6-month follow-up, recruiting prefrail or frail people aged ≥70 years living at home from three European countries (N=83), and B) a validation study in usual clinical practice called INTEGRACAM, conducted in Madrid, Spain, under a quasi-experimental before-after design that mirrors the criteria of POSITIVE. Both components utilized a technological platform for comprehensive patient assessment and functional status monitoring, delivering personalized interventions including VIVIFRAIL physical exercise programs, nutritional recommendations, and medication adjustments according to STOPP-START criteria. Usability and user experience were measured using the System Usability Scale (SUS) and the User Experience Questionnaire (UEQ-S), alongside frailty (Fried criteria and FTS-5 score) and functional status (SPPB and WHODAS). Results from POSITIVE (average age 76.7 years; 76.8% female) indicated significant improvements after six months in frailty status (FTS-5: -5.43 points in the intervention group vs. usual care; p=0.008; Fried´s criteria-FFP: -0.4 in IG vs UC p=0.087), SPPB (+1.07 points; p= 0.022) and WHODAS (0.22, p= 0.09). INTEGRACAM showed similar findings with participants (n=51; mean age 78.1±5.3 years) demonstrating improvements in SPPB (+1.03±1.38; p-value <0.001), FTS5 (-3.4±4.11; p-value <0.001), and FFP (-0.4±1.03; p-value 0.028) after 6 months. Patients rated usability as 87/100, surpassing the optimal threshold value (68/100). Regarding the UEQ-S, hedonic quality improved throughout the project (2.44 vs 2.02, p=0.031), both in patients and professionals.

